# Spatial–temporal contrast sensitivity of the eye alignment reflex

**DOI:** 10.1038/s41598-022-23753-1

**Published:** 2022-11-14

**Authors:** Deepa Dhungel, Scott B. Stevenson

**Affiliations:** University of Houston College of Optometry, 4401 Martin Luther King Blvd, Houston, TX 77204 USA

**Keywords:** Oculomotor system, Visual system

## Abstract

The binocular alignment of the eyes involves both voluntary and reflexive mechanisms, but little is known about the visual input and neurological pathway of the reflex component. Our studies examined the role of spatiotemporal frequency and contrast in the control of reflex eye alignment, and compared the contrast sensitivity of the alignment reflex with psychophysical contrast sensitivity. We measured the contrast sensitivity of vertical disparity-driven vergence eye movements in response to bandwidth filtered static or 6 Hz counterphase flickering noise and measured psychophysical detection sensitivity for the same stimuli. Contrast thresholds for producing a detectable vertical alignment change (measured with nonius lines) were determined using a staircase method for 7 spatial frequencies [0.25–16 cycles per degree] and 3 vertical disparities [5, 10, and 30 arcmin] in 7 adults with normal or corrected to normal vision. The main findings of this study are, (1) the vertical alignment reflex had overall relatively high contrast sensitivity, comparable to but somewhat less than visual detection thresholds, (2) the most effective stimulus spatial frequency scaled in inverse proportion to the disparity being stimulated, and (3) unlike psychophysical contrast sensitivity, the eye alignment reflex contrast sensitivity was not improved by flickering low spatial frequencies.

## Introduction

Proper eye alignment is an important factor in maintaining comfortable binocular vision. Disparity vergence is a visually-driven disjunctive eye movement that corrects misalignments of the eyes in horizontal, vertical, and torsional directions^1,2^. While horizontal vergence has been studied extensively, vertical vergence has not been studied in detail. Our studies examined the role of contrast in the control of vertical eye alignment, and how the vergence contrast sensitivity compares with psychophysical contrast sensitivity. Because the pathway for vertical alignment control is uncertain, it is interesting to know if visual responses in the two cases are similar. Vertical vergence is a small, slow, disjunctive, reflexive eye movement made in response to vertical image misalignment (retinal disparity)^3^. The typical maximum response is about 1.5$^{\circ }$–3$^{\circ }$ (3–5 Prism Diopters) of eye rotation^4^, and most responses are considerably smaller. Measurement of vertical vergence is accomplished with a sensitive eye tracker or with nonius alignment judgments.

Experiments with sensitive eye tracking systems have shown that vertical eye alignment has relatively low variability during fixation (rms 2–3 arcmin)^5,6^, and is maintained even across large eye movements^7^. A small vertical disparity change produces a realignment of the eyes with an onset latency as low as 100 ms^8^, but responses to larger disparities can take a second or more to complete^9^. Vertical vergence exhibits characteristics of a visually driven reflex. The effort to make, or to resist a change in eye alignment under imposed vertical disparity has little or no effect on the response^9^. Likewise targets that are intentionally tracked vs ignored produce similar responses to added vertical disparity jitter^8^, provided they have similar eccentricity. In our experience, unlike horizontal vergence, vertical vergence cannot be produced voluntarily, with no stimulus. The visual inputs to the alignment reflex have received relatively little attention. Responses to dynamic, random dot stimuli underscore the truly binocular nature of the response: vergence movements occur even in the absence of any monocular information that might drive independent monocular tracking^10^. Comparison of targets in the periphery vs fovea found they were equally effective if the target area was scaled for cortical magnification^10^. In recent experiments with a sensitive dPi^11^ eye tracker^12,13^, we found that the vertical vergence response was robust (though small) even down to near-threshold contrast for mid-ranged spatial frequencies. Here we extend those investigations with a nonius offset discrimination task. It is relatively low tech and inexpensive but comparable to a dPi eye tracker in precision. The subject judges the relative perceived position of two lines, one in each eye, similar to a vernier alignment task^14^. However, the lines are presented dichoptically so that the signals originate from different eyes and vertical misalignment of the eyes produces a perceived offset in the nonius lines. Whatever the amount of stimulating disparity, if the eyes realign by even a few arcmin, the subject can detect it as a nonius offset and identify the direction of disparity. The purpose of the first in the series of five experiments was to establish that nonius offset discrimination task can be used for measuring vertical eye alignment in our subjects and to determine the sensitivity of the technique to small offsets. We measured the nonius offset discrimination threshold by presenting various physical offsets between the nonius lines randomly while the vertical disparity of the background remained zero.

Contrast sensitivity functions (CSFs) has a dependence on luminance flicker. Steady or slowly changing grating targets produce a bandpass CSF, with a roll off at both high and low spatial frequencies relative to the peak around 2–4 cpd^15^. Counterphase flickering gratings produce a low pass CSF, with a high frequency roll off but no low frequency roll off. In our second and third experiments we measured the contrast sensitivity of vertical eye alignment with a nonius offset discrimination task across various spatial frequencies in response to steady (Exp 2) and to 6 Hz counterphase (Exp 3) noise patterns. To find out if the response of vertical eye alignment is similar to the perceptual responses, we measured the psychophysical CSFs in our fourth and fifth experiments using the steady and counterphase flicker noise patterns, respectively. We found that psychophysical CSF changed as expected, increasing for low spatial frequency stimuli with the addition of counterphase flicker. Vertical vergence CSF, in comparison, showed no change with counterphase flicker, being bandpass with both steady and counterphase flickering targets. Disparity sensitivity in visual mechanisms has a close relationship to spatial frequency tuning^16^, and responses to 5, 15 and 30 arcmin stimuli showed that the most effective spatial frequency shifted lower for larger disparity.

## Results

### Nonius offset discrimination

**Figure 1:**
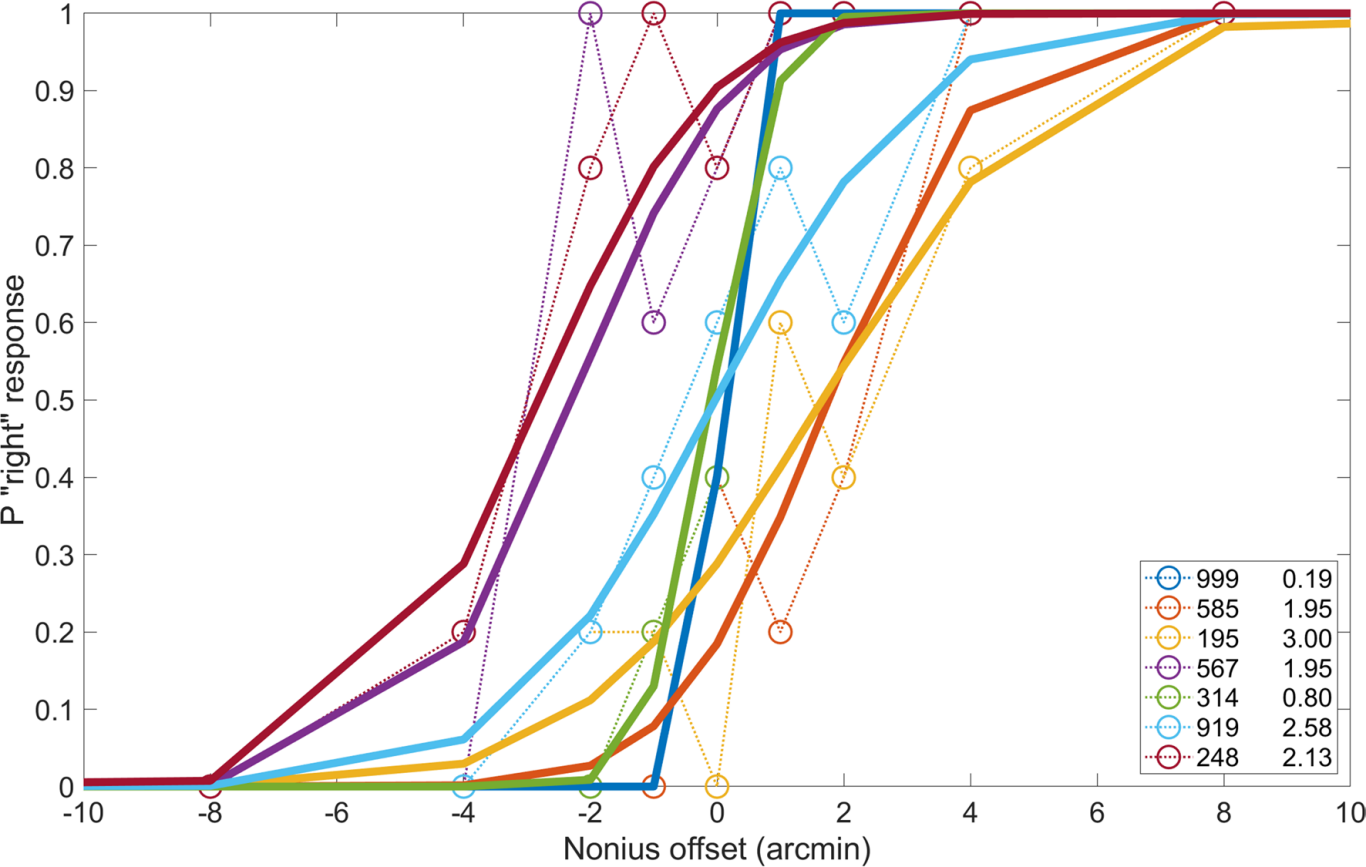
Nonius offset discrimination curves for seven subjects. Positive values of offset on the horizontal axis indicate that the right nonius line was shifted up relative to the left line, negative values indicate that the left line was shifted up. The vertical axis shows the probability that subjects indicated that the right line appeared higher. The precision of the nonius alignment judgments is indicated by the standard deviation of the best fitting cumulative normal curve, and these values are shown in the legend for each subject. Raw data (circles and thin lines) are shown along with fitted cumulative Gaussian functions (solid lines). Subjects could reliably detect offsets of 3 arc minutes or less. Subjects 999 and 585 are the authors.

To estimate the precision of the nonius method, we added small offsets to one of the lines and asked subjects to report which appeared shifted upward. We found that some subjects can reliably detect offsets as small as 1 arcmin. The results from this experiment are shown in Fig. 1. The plot shows curves in different colors representing the nonius offset discrimination performance for each subject. The percentage of “right” responses, meaning the right hand line was perceived to be higher than the left line, is plotted against disparity, the shift of the image in one eye relative to the other. Fitted cumulative normal curves are plotted in solid lines, along with the individual responses in dotted lines with symbols. The Standard Deviation (SD) values of the fit listed on the bottom right of the plot can be used as an estimate of the precision of this method for detecting shifts in eye alignment. The nonius discrimination threshold ranged from less than 1 to around 3 arcmin, and the average for 7 subjects was 1.8 arcmin.

The lateral shifts in the individual curves indicate a constant error or nonius bias, and these were also quite small. From these results we are confident that subjects are able to report relatively small shifts in eye alignment with this method, and that its precision is on par with the best available eye trackers.

### Vertical vergence CSFs for steady vs 6 Hz flicker noise

**Figure 2:**
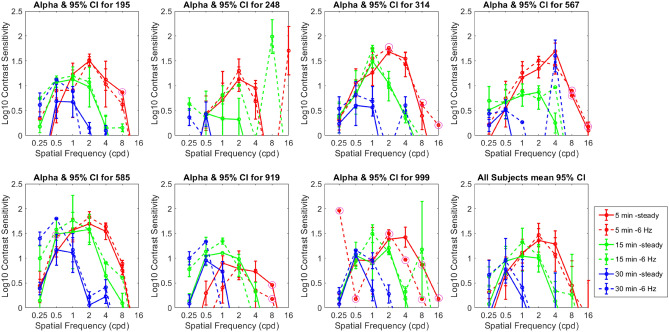
Vertical vergence CSFs for static vs 6 Hz flickering spatially filtered noise. The noise had a bandwidth of 1 octave and included all orientations. The Spatial Frequency values in the plot indicate the center of each noise band. Larger disparities showed a loss of response at high spatial frequencies, but similar responses at low. The CSFs for flickering and static noise are similar in shape for a given disparity. Responses at 16 cpd and above were absent or unreliable. For the most effective stimulus, a 2 cpd pattern at 5 arcmin, the peak sensitivity represents a contrast threshold of around 3%. The magenta circles around the data points indicate that our bootstrap method did not produce error bars because the beta values of the Weibull fit were too steep.

Contrast sensitivity for vertical vergence was measured using the nonius method to find the lowest contrast that would produce a detectable shift of the eyes, allowing the subject to identify the direction of disparity based on the direction of the offset. Stimuli were spatially filtered noise patterns (Fig. 6) with a narrow (one octave) band of spatial frequencies and one of three disparities (± 5, 15, and 30 arcmin ). In separate experiments with the same subjects, we measured sensitivity with steady or 6 Hz counterphase flickering noise patterns. Results are plotted in Fig. 2 for 7 subjects. Contrast sensitivity is plotted separately for each subject, with the subject average shown in the bottom right panel. The disparity condition is color coded. The CSFs for flickering and steady noise were similar in most cases for a given disparity. The sensitivity at the three disparities was similar for low spatial frequencies, but there was a loss of higher spatial frequency sensitivity as the disparity got larger. CSFs of the smallest disparity condition (5 arcmin) included responses at a wider range of spatial frequencies than the higher disparities. It is interesting to note that the peak of the CSFs shifted systematically towards the left as the disparity increased. The CSF for 5 arcmin peaks at around 3 cpd, 15 arcmin at around 1 cpd, and 30 arcmin at around 0.50 cpd.

### Psychophysical CSFs for steady vs 6 Hz flicker noise

**Figure 3:**
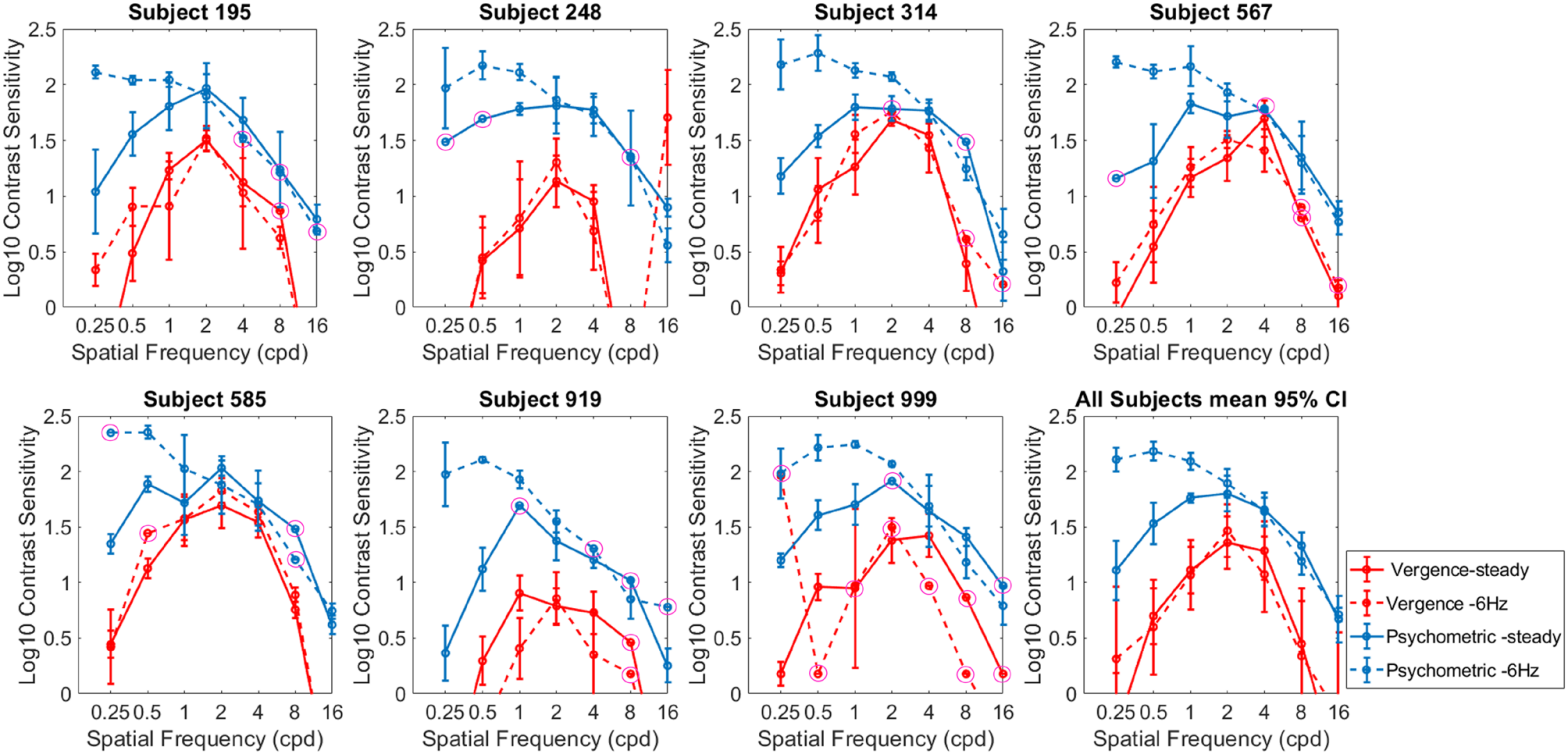
Comparison of vertical vergence CSFs (red) vs psychophysical CSFs (blue) at 5 arcmin disparity. The shape of the vergence CSF was similar to the psychophysical CSF, but with overall lower sensitivity. Flicker showed a strong effect at low spatial frequencies in the psychophysical data (blue dashed curves), but not in the vergence data (red dashed curves).

For comparison to the vertical vergence data, we also measured our subjects’ ability to detect the steady and flickering filtered noise patterns. The disparity was 5 arcmin. The blue solid and dashed curves in Fig. 3 show the Psychophysical CSFs for steady noise and the 6 Hz flicker noise respectively, and the vergence data for 5 arcmin are replotted in red for comparison. As expected, the effect of flicker was strong on the contrast sensitivity at low spatial frequencies in the psychophysical CSFs. The addition of flicker changes the bandpass shape of the CSF into a low pass shape, consistent with previous work^15^. This change did not occur for the vergence CSF.

### Vertical vergence CSFs vs psychophysical CSFs

**Figure 4:**
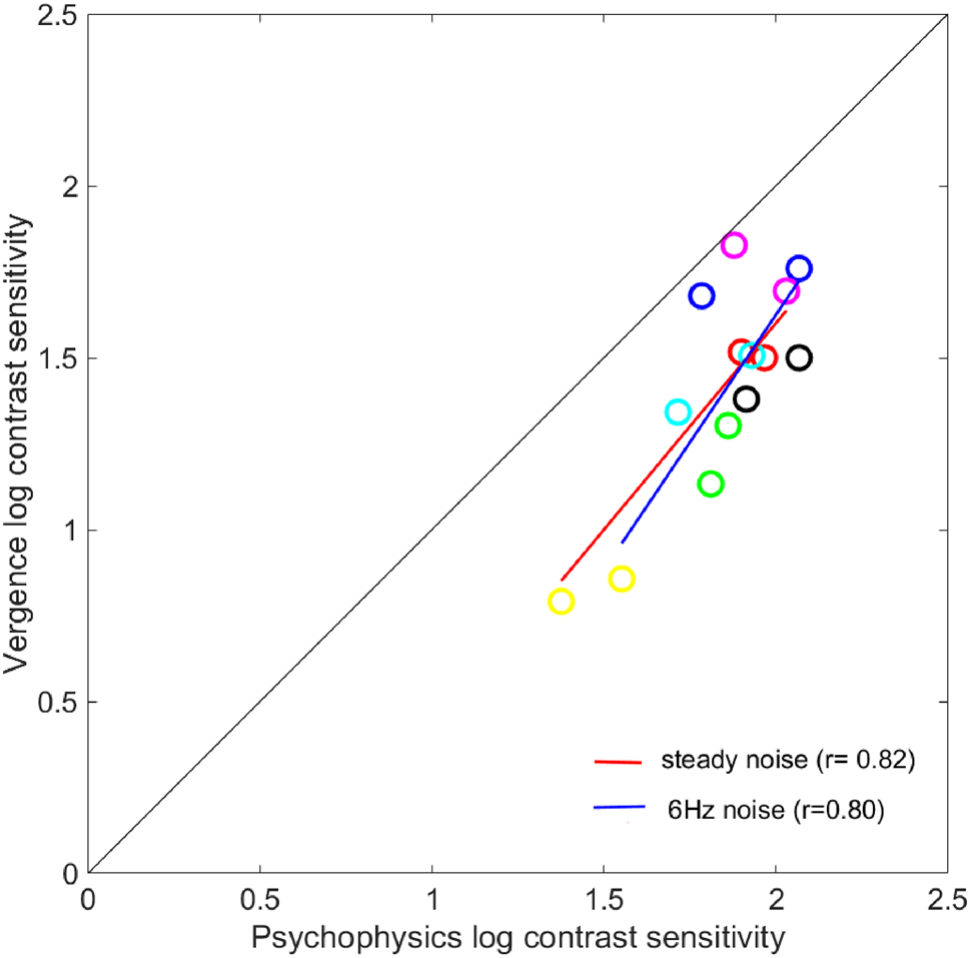
Scatter plot showing peak vergence contrast sensitivity vs psychophysical contrast sensitivity at 2 cpd. Circle color represents the subjects, there are two circles for each subject, one that is for steady and one for flicker noise, red slanted line represents regression line for steady noise and blue line represents 6 Hz noise regression line, the solid dark slanted line represents a 1:1 line.

For the steady noise condition, the shape of the vergence CSF was similar to the psychophysical CSF, but with overall lower sensitivity (Fig. 3). Most subjects did not respond to 16 cpd even at 100% contrast in the vergence experiments. Flicker showed minimal or no effect at low spatial frequencies in the vergence data whereas the effect of flicker on psychophysical data was strong. Comparing the two types of response, a spatial frequency of 2 cpd showed the least difference in sensitivity between the two methods, and results across subjects showed a good correlation between them. Figure 4 is the scatter plot of the vertical vergence contrast sensitivities of each subject represented by different colors, plotted against psychophysical contrast sensitivities. We found that there was a strong correlation of Psychophysical contrast sensitivity and vertical vergence contrast sensitivity for both steady noise (r = 0.82) and 6 Hz noise(r = 0.80) data. Most subjects show a similar change on both measures when going from steady to flicker. The contrast sensitivity of vertical vergence is therefore a reasonably good predictor of psychophysical contrast sensitivity at 2 cpd spatial frequency.

## Discussion

The measurements we report here rely on the nonius technique for measurement of small vergence changes. Nonius, or dichoptic Vernier lines, are the method used by clinicians to measure small binocular errors in fixation, termed fixation disparity^17^. This test presents a binocular fusion target along with monocularly seen left eye and right eye nonius lines. Small changes in vergence produce noticeable shifts in the relative location of the left and right eye lines, even while the binocular target remains fused.

Experiments by Duwaer and Van Den Brink^18^ showed that observers could detect nonius offsets for vertical vergence responses as small as 1 arcmin, well below the diplopia detection threshold of 10–20 arc min. Experiments by McKee and Levi^14^ found that monocular vernier thresholds were much better than dichoptic vernier thresholds. They estimated that the difference could be accounted for by fluctuations in horizontal vergence during fixation. Some experiments have called into question whether nonius line judgements might be influenced by a change in retinal correspondence, such that the perceived offset may not be due only to vergence changes^19,20^ (but see also^21,22^). In our study, nonius judgements are likely limited by the fluctuations of vertical eye alignment that occur during fixation. Over a period of several seconds, a fixating observer will typically have vertical vergence fluctuations of about 2 arc min RMS^5^. Our subjects typically did this well or better at detecting small offsets in the nonius lines Fig. 1. If binocular correspondence shifted in the direction of the background disparity and changed the apparent nonius offset, then our method may underestimate the amount of vergence occurring and the contrast threshold for initiating vergence. We consider this unlikely.

The results from our experiments also show that the contrast sensitivity of the vertical eye alignment reflex can be readily measured with the nonius offset task. The measured contrast sensitivity parallels the contrast sensitivity measured psychophysically with steady noise patterns, but with slightly lower sensitivity. Some subjects showed a small bias in their nonius offset judgments, which might have elevated thresholds slightly. Although we measured this nonius bias in our first experiment, we did not correct for it in the subsequent experiments because it was less than 3 arc minutes. While small offsets (less than 2 arcmin) might have been missed by subjects, it is unlikely that this significantly reduced the measured contrast thresholds. It is possible that with a more sensitive technique, such as a binocular Tracking Laser Ophthalmoscope^5^, eye alignment responses to even lower contrasts or to higher spatial frequencies might be detectable.

A second main finding from our experiments was that adding flicker to our stimuli did not improve the low spatial frequency response in the vertical eye alignment system. In psychophysical measures of contrast sensitivity, there is a pronounced improvement in sensitivity to low spatial frequencies when the stimulus is flickered, particularly for frequencies around 6–8 Hz^15,23,24^. Research into the basis of this effects suggests that the human CSF is determined by a large number of spatial frequency channels^25^and a smaller number of temporal frequency channels, sometimes described as sustained vs transient mechanisms^26^. Our findings suggest that the control of vertical eye alignment may have different inputs compared to the pathway leading to perception, or that only a subset of visual mechanisms (e.g. sustained but not transient) in a common pathway are driving the motor response. It should be noted, however, that the vertical vergence response requires localization as well as detection. It would be interesting to make a comparison of contrast thresholds for localization vs. detection in a different task, such as vernier acuity. Except for this lack of flicker effect, the response of the eye alignment system at medium and high spatial frequency showed sensitivity similar to that measured psychophysically for grating detection, and the two systems showed the same CSF shape when tested with non flickering targets.

A third main finding from our experiments concerns the interaction of disparity and spatial frequency. Previous experiments^27^ and models^16^ have proposed a correlation between the optimum disparity for response and the spatial frequency tuning of disparity detectors. So called disparity energy models^28,29^ in which responses from the left and right eye detectors are multiplied, predict a maximum response when the disparity is 1/4 of a cycle (90 degrees phase) of the peak spatial frequency. These models were proposed to account for stereoscopic depth perception from horizontal disparity, but the original Marr and Poggio^16^ model also proposed a course to fine control of horizontal convergence eye movements that assumed a size-disparity correlation in disparity processing. Some counter evidence to the proposed encoding scheme was reported by Badcock and Schor, who showed that the disparity discrimination performance was best for high spatial frequencies, even if the discriminated targets both had relatively large standing disparity^30^. Similarly, Yang and Blake^31^, measured monocular noise masking of a stereo target in various spatial frequency combinations of target and mask, and found a lack of support for a strict correlation of size and disparity. Given that the vertical eye alignment system lacks the perceptual and volitional characteristics of horizontal eye alignment control, we were interested to see if a size-disparity correlation would also occur for vertical disparity detection. We used three different fixed disparities in our vertical alignment experiments and we noticed a systematic shift in the most effective spatial frequency (defined as the lowest contrast needed to move the eyes, at the peak of the CSF). We found that as the disparities got smaller, peak contrast sensitivity shifted to higher spatial frequencies. The size-disparity correlation we found is similar to that found by Smallman and Mcleod^27^ for psychophysical detection of low contrast sine gratings with horizontal disparity. These results suggest that a model similar to Marr and Poggio’s course-to-fine approach may account for disparity processing in the reflexive control of eye alignment, even if it is not a complete explanation for the perception of depth from disparity.

**Figure 5:**
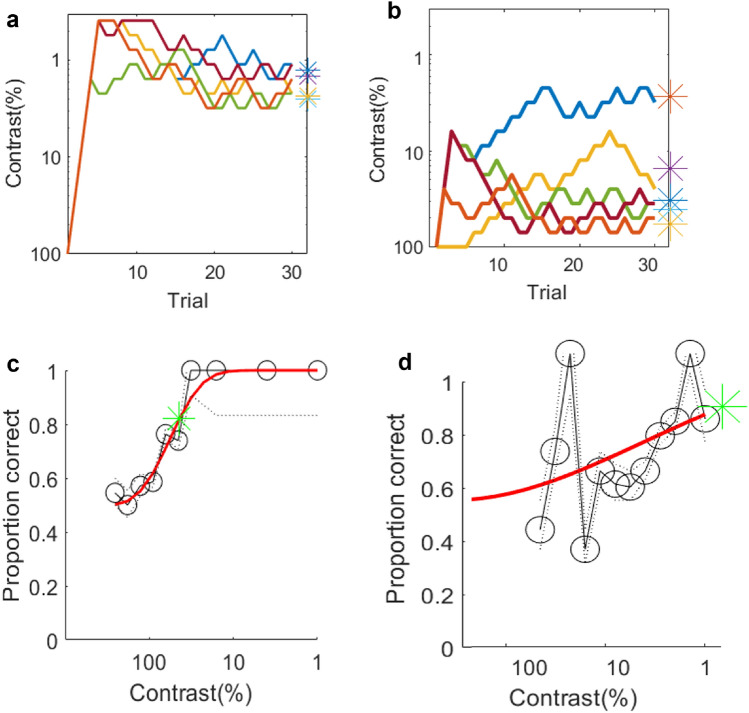
Examples of the staircases in the top row and their corresponding Weibull fits in the bottom row, a good weibull fit (**a**,**c**) and a poor fit (**b**,**d**). The different colored curves in the plots **a** and **b** represent five staircases from one subject for the same spatial frequency and disparity condition. Response data from trials in all five staircases were combined into a single psychometric function and fit with a Weibull^32^ to estimate the alpha value, corresponding to 82% correct performance. The red curves on the plot in the bottom row show the Weibull fit to the raw data, shown with the grey curves and circular symbols. The green asterisks are the alphas of the fits and were used as threshold estimates. The alpha in the poor fitting staircase corresponds to a contrast greater than 100%. Cases where a fit was unreliable are marked in the plots of Figs. 2 and 3 as open magenta circles.

When we compare the CSFs of vertical eye alignments and psychophysical detection CSFs (blue vs red curves in Fig. 3), the eye alignment response has lower sensitivity but a similar shape. As a result, high spatial frequencies that were perceptually above threshold did not produce detectable offsets in the nonius lines, and it is unclear if these spatial frequencies are used for eye alignment or not. The staircase functions in these conditions often hit the maximum of 100% contrast, and fitted Weibull functions to the responses produced threshold estimates beyond 100% that were therefore unreliable. Figure 5 shows an example each of a good and a bad Weibull fit and the corresponding staircases.

In summary, the CSF of vertical eye alignment control showed a shape similar to the psychophysical CSF, with comparable overall sensitivity. The most significant difference occurred with flickering targets at low spatial frequency, where flicker had no effect on the sensitivity of the eye alignment response. Small disparities produced responses over a larger range of spatial frequency than large disparities, with support for a size disparity correlation in the mechanisms producing the response. Finally, we have established that a simple, easily accessible, low-tech nonius task can be used to measure binocular contrast sensitivity of vertical eye alignment with high precision and sensitivity. It may be useful in some cases as the basis for a quick screening tool for assessing binocular vision and contrast sensitivity simultaneously.

## Methods

### Subjects

Seven subjects (including 2 authors), with normal or corrected to normal visual acuity and binocular vision were enrolled in the study. Best-corrected distance visual acuity of 20/25 or better in each eye, assessed using MAR visual acuity chart, and presence of coarse stereo acuity using a TNO stereo acuity test were the inclusion criteria. Subjects with presence of amblyopia, constant strabismus, and previous history of ocular surgeries were excluded from the study. All the experimental protocols were approved by the Institutional Review Board at the University of Houston. The research was carried out in accordance with the Tenets of the Declaration of Helsinki. Written consent was obtained from each subject after explaining the experimental procedures. Subjects were compensated for their time.

### Stimuli and testing apparatus

**Figure 6:**
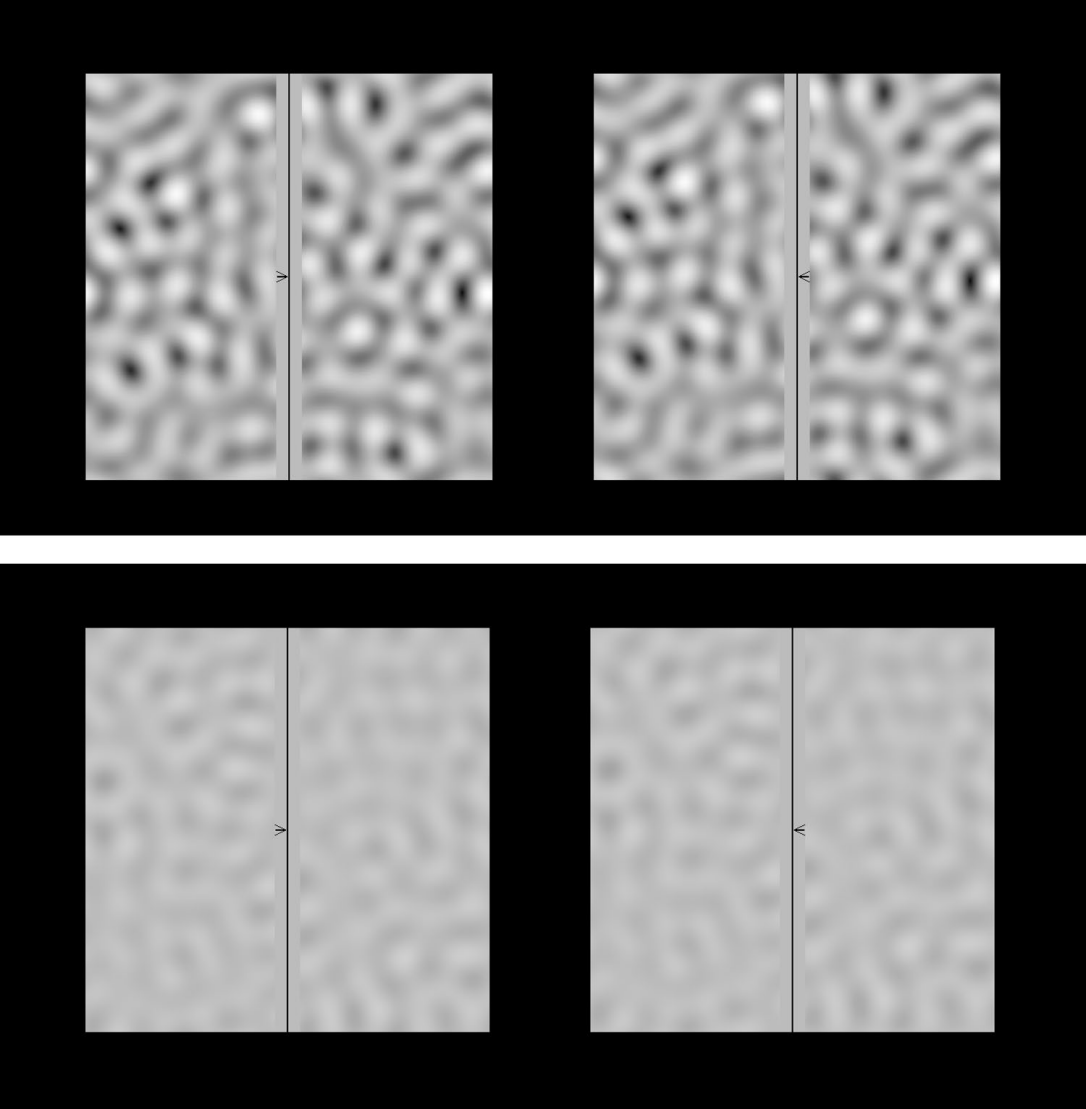
Examples of the stimuli, a high contrast (top) and a low contrast (bottom) one octave bandwidth noise pattern. The arrow heads on each sides of the central vertical lines are the nonius lines. Free fusing the two images makes it possible to appreciate the nonius offset.

**Figure 7:**
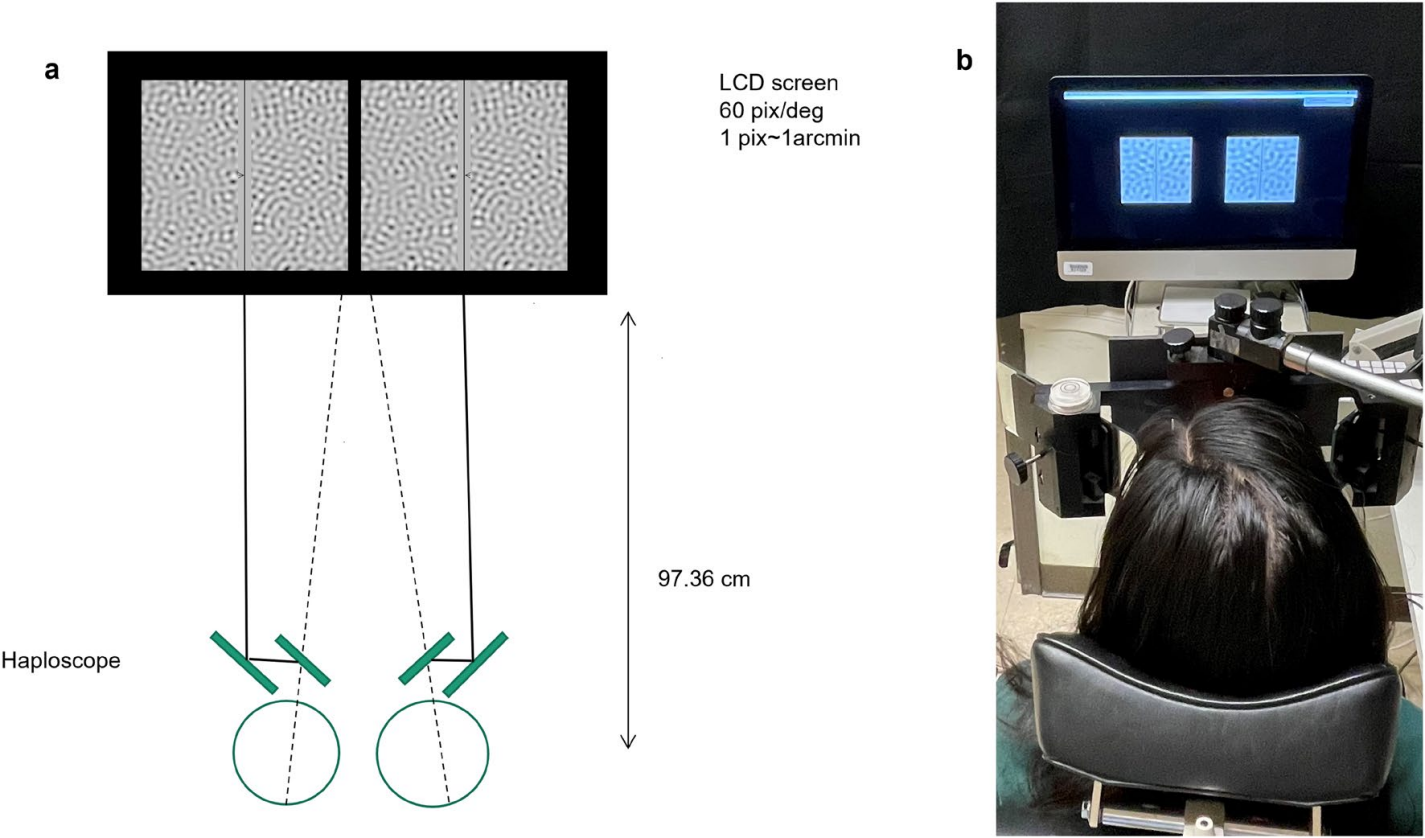
(**a**) Schematic diagram of the experimental setup, figure is not drawn to scale. (**b**) Subject viewing stimuli through the haploscope on a monitor screen with the head positioned on the head rest. The convergence angle of the eyes was adjusted to match the optical distance to the targets for comfortable fusion. Trials were run in dim room light and the subject’s view was masked off to ensure that each eye saw just one image with a dark surround.

Stimuli were spatially-filtered noise patterns, presented steadily or with 6 Hz counter-phase flicker (Fig. 6). The filter was one-octave wide rectangular bandpass and spatial frequencies were the middle of the range. Unlike a repeating pattern stimulus such as a sine wave grating, a bandpass filtered noise pattern includes a small range of spatial frequencies and multiple orientations, thus greatly reducing the probability of matching ambiguity. Contrast thresholds were determined for 7 spatial frequencies [0.25, 0.50, 1, 2, 4, 8, and 16 cpd] and 3 disparities [5,15 and 30 arcmin] using a staircase method. A MATLAB program was used to generate the stimulus, display it on the screen, and store the responses. The stimuli were presented dichoptically on a gamma-corrected screen with a mean luminance of 130 cd/m$^{2}$ and a resolution of 60 pixels per degree. A schematic diagram of the experimental setup is shown in Fig. 7(a), Fig. 7(b) shows a subject performing the task. We used 8 bit grayscale with dithering to produce contrast levels as low as 0.4%. Stimuli consisted of a stereo pair of 512 $\times$ 512 pixel images on a dark background, presented side by side on a single screen and viewed through a four mirror haploscope. Each image had a 32-pixel gray strip running vertically through its center, with a 2 pixel dark vertical line in the middle. The central vertical line served as a target to maintain a clear focus and steady horizontal fixation. A horizontal arrow 16 $\times$ 2 pixel at the center of the image towards the left on the left eye image and towards the right on the right eye image served as a nonius line to give feedback to the subject about the eye alignment. Vertical disparity was introduced into the noise pattern by shifting the right eye noise either up or down, while leaving the frame and nonius lines in place. When fusing the stereogram, the relative perceived position of the horizontal arrows along the vertical direction is referred to as the nonius offset, and it indicates the relative alignment of the eyes. When subjects perceive the right eye nonius as higher, it means that the right eye’s pointing direction is slightly lower than the left eye, indicating that the eye alignment has changed. If the shift in eye alignment reliably matches the disparity direction presented, it indicates that the pattern is effective in controlling eye alignment.

### Experimental procedures

#### Experiment 1: nonius offset discrimination experiment

Subjects were seated on a chair with a headrest to minimize head movement, with the haploscope in front of them supported by a stand off to one side. A standard keypad was used to record the responses. The experiments were carried out in a dark room with the computer screens being the primary source of light. At the beginning of the experiment, a practice block was run which helped the subjects to get familiar with the task and determine their nonius offset detection performance. Rather than shift the disparity of the noise, the noise disparity was kept at zero and the nonius target itself was redrawn with an offset [± 0, ± 1, ± 2, ± 4, ± 8, ± 16, ± 32 arcmin]. The sign of the offset indicates if the right or left eye target was higher. The trial sequence and timing used were the same as for the following experiment, except that the contrast of the noise background was set to zero for the 2 s period before the nonius appeared and the offset judgement was made. The frame of the display and vertical line served as the only fusible contours during this period. The subjects responded to which of the nonius appeared higher by pressing “1” if the left nonius line was higher or “2” if the right was higher. A cumulative Gaussian was fit to the psychometric functions, with the SD of the underlying Gaussian serving as an estimate of offset detection precision. All subjects show precision of 3 arc minutes or better, with 1 arc minute being typical.

#### Experiment 2: steady noise nonius discrimination experiment

**Figure 8:**
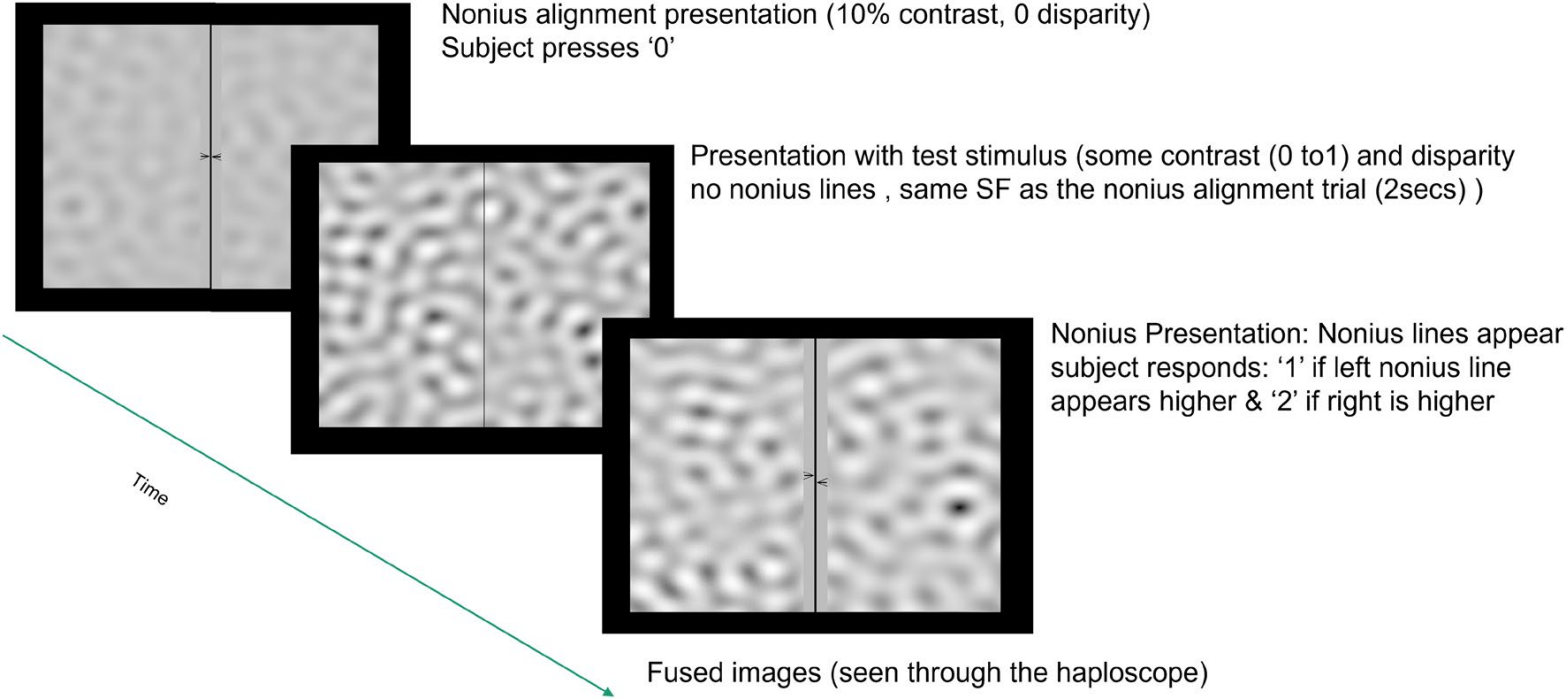
Sequence of events in a trial in the steady noise nonius discrimination experiment. After visually confirming that the nonius marks were aligned , subjects hit a key and viewed the disparate stimulus without nonius lines for 2 s to allow the eyes time to respond. The nonius marks then reappeared and the subject responded with another keystroke to indicate the perceived offset.

After the completion of the nonius judgement trials, we proceeded to measure contrast thresholds for producing an alignment change using noise patterns with vertical disparity. The nonius lines were always drawn at the center of the image, so any perceived offset would reflect a change in eye alignment. A two alternative forced choice (2AFC) simple staircase (1 up, 2 down) was run for every combination of the 7 spatial frequency and 3 disparity conditions, with five repetitions. Each staircase started at a high contrast level, the contrast dropped with two correct responses in a row, and increased with each incorrect response until 30 presentations. Contrast reduced by a factor of 4 until the first error, then by a factor of square root of 2. As in the first experiment, every trial started with a nonius alignment presentation with noise patterns at 10% contrast and zero disparity. When the nonius lines appeared aligned, the subject pressed a ‘0’ to initiate the trial. The noise pattern with disparity was shown for 2 s, after which nonius lines appeared. The subject indicated the direction of nonius offset with a keystroke. Subjects got audio feedback, indicating correct or incorrect responses. Figure 8 summarizes the sequence of events in a trial in the static noise nonius discrimination experiment.

#### Experiment 3: 6 Hz flicker noise nonius discrimination experiment

The experimental setup and the procedures were similar to the previous setup, except the noise patterns were counter-phase flickered at 6 Hz. The frame and nonius lines did not flicker. Experimental conditions were otherwise the same as Experiment 2.

#### Experiment 4: psychophysical steady noise contrast detection experiment

For comparison to the eye alignment results, we measured psychophysical detection thresholds for the same stimuli, keeping all conditions the same as far as possible. We used a temporal, 2 interval forced choice procedure for the subject to identify if the first or the second interval contained a noise pattern in a 200 ms exposure time for each interval. In both nonius method and psychophysical method, increasing duration might increase the sensitivity. If the stimulus was presented for too long in the psychophysical task, it could allow the eyes to make vergence movement and thus change the retinal disparity. If the vertical vergence threshold was lower than the psychophysical threshold, having a longer duration would allow the detection of the nonius shift and thus provide a cue for detecting the pattern rather than contrast. We chose a duration that we thought was long enough to be longer than the temporal integration time for contrast detection but still shorter than the typical vertical vergence reaction time.

**Figure 9:**
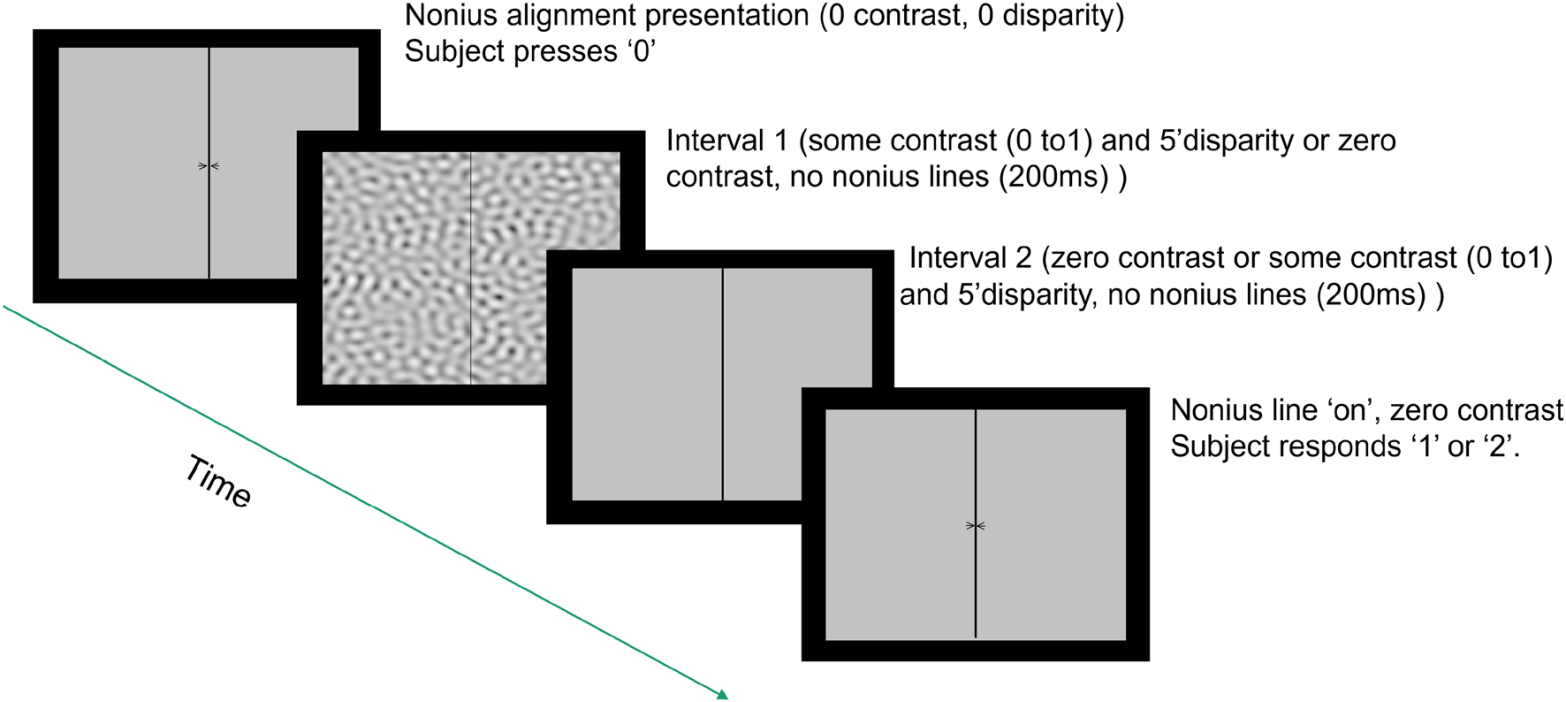
Sequence of events in a trial in the psychophysical steady noise contrast detection experiment. Stimuli were presented with fixed vertical disparity, and subjects indicated which of the two intervals had the stimulus. Presentation was brief to prevent a vergence response so that retinal disparity was consistent from trial to trial.

Sequence of events in a trial in the psychophysical contrast detection experiment is shown in Fig. 9. Contrast thresholds were measured with the same staircase rule as in the previous experiments, and for the same values of spatial frequency. Vertical disparity was fixed at 5 arcmin.

#### Experiment 5: psychophysical 6 Hz flicker noise contrast detection experiment

The experimental setup and the procedures were same as in the psychophysical static noise contrast detection experiment except the stimuli were 6 HZ flicker-noise patterns, as used in Experiment 3.

### Statistical analysis

Staircase data were analyzed using custom software written in MATLAB 2019b. The performance across five staircase runs was tallied to find the proportion correct for each contrast. These psychometric functions, representing 150 trials altogether, were fit with a Weibull^32^ function in order to estimate the contrast that produced 82% correct performance. Confidence intervals were determined with a bootstrapping method.

## Data Availability

The datasets generated during and/or analysed during the current study are available from the corresponding author on reasonable request.
