# Author Correction: Spatial–temporal contrast sensitivity of the eye alignment reflex

**DOI:** 10.1038/s41598-023-40100-0

**Published:** 2023-08-08

**Authors:** Deepa Dhungel, Scott B. Stevenson

**Affiliations:** University of Houston College of Optometry, 4401 Martin Luther King Blvd, Houston, TX 77204 USA

Correction to:* Scientifc Reports* 10.1038/s41598-022-23753-1, published online 14 November 2022

The original version of this Article contained an error.

In the Results section, under the subheading ‘Vertical vergence CSFs for steady vs 6 Hz flicker noise.’,

“It is interesting to note that the peak of the CSFs shifted systematically towards the left as the disparity decreased.”

now reads:

“It is interesting to note that the peak of the CSFs shifted systematically towards the left as the disparity increased.”

The original article has been corrected.

